# Modulators of hormonal response regulate temporal fate specification in the Drosophila brain

**DOI:** 10.1371/journal.pgen.1008491

**Published:** 2019-12-06

**Authors:** Giovanni Marchetti, Gaia Tavosanis

**Affiliations:** 1 Dynamics of neuronal circuits, German Center for Neurodegenerative Diseases (DZNE), Germany; 2 LIMES-Institute, University of Bonn, Germany; Duke-NUS Medical School, SINGAPORE

## Abstract

Neuronal diversity is at the core of the complex processing operated by the nervous system supporting fundamental functions such as sensory perception, motor control or memory formation. A small number of progenitors guarantee the production of this neuronal diversity, with each progenitor giving origin to different neuronal types over time. How a progenitor sequentially produces neurons of different fates and the impact of extrinsic signals conveying information about developmental progress or environmental conditions on this process represent key, but elusive questions. Each of the four progenitors of the *Drosophila* mushroom body (MB) sequentially gives rise to the MB neuron subtypes. The temporal fate determination pattern of MB neurons can be influenced by extrinsic cues, conveyed by the steroid hormone ecdysone. Here, we show that the activation of Transforming Growth Factor-β (TGF-β) signalling via glial-derived Myoglianin regulates the fate transition between the early-born α’β’ and the pioneer αβ MB neurons by promoting the expression of the ecdysone receptor B1 isoform (EcR-B1). While TGF-β signalling is required in MB neuronal progenitors to promote the expression of EcR-B1, ecdysone signalling acts postmitotically to consolidate theα’β’ MB fate. Indeed, we propose that if these signalling cascades are impaired α’β’ neurons lose their fate and convert to pioneer αβ. Conversely, an intrinsic signal conducted by the zinc finger transcription factor Krüppel-homolog 1 (Kr-h1) antagonises TGF-β signalling and acts as negative regulator of the response mediated by ecdysone in promoting α’β’ MB neuron fate consolidation. Taken together, the consolidation of α’β’ MB neuron fate requires the response of progenitors to local signalling to enable postmitotic neurons to sense a systemic signal.

## Author summary

Throughout the development of the central nervous system (CNS), a vast number of neuronal types are produced with striking precision. The unique identity of each neuronal cell type and the great cellular complexity in the CNS are established by intricate gene regulatory networks. Disruption of these identity programs leads to neurodevelopmental disorders and defects in cognition. Here, we report an important regulatory mechanism involved in consolidating neuronal fate. We show that during brain development local signalling, derived from interactions between glial cells and neuronal progenitors, is required to promote the expression of a hormone receptor in immature neurons. The perception of a systemic hormonal cue in those postmitotic neurons is fundamental for the consolidation of their neuronal fate. In this context, we additionally uncover an intrinsic regulatory mechanism that coordinates the hormone response to maintain the final neuronal fate.

## Introduction

The central nervous system displays great diversity of neuronal cell types, which are assembled into neural circuits to serve brain functions [1]. This neuronal variety is generated during development when neural stem cells and progenitor cells generate neurons of distinct fates in a birth order dependent manner. An initial period of neurogenesis is followed by a phase of sequential specification in which different types of neurons are produced during defined time windows [1]. Pioneer studies performed in the *Drosophila* embryo have uncovered that temporal fate specification is governed by the sequential expression of transcription factors in the progenitors, providing temporal identity to their daughter cells [2]. However, complementary studies in vertebrate models indicated that, together with intrinsic factors, external cues are required to define the fate competence of neuronal progenitors [3, 4]. This discrepancy was recently challenged as recent studies in *Drosophila* postembryonic lineages revealed the importance of external cues in ensuring the proper temporal generation of neuronal diversity [5, 6]. In those systems extrinsic factors seem to display complex interdependent functional relationships with intrinsic transcriptional programs that are just starting to be elucidated [5, 6]. Hence, a fate program involving both external and intrinsic factors appears to be conserved and essential during brain development to generate neuronal variety.

**Fig 1:**
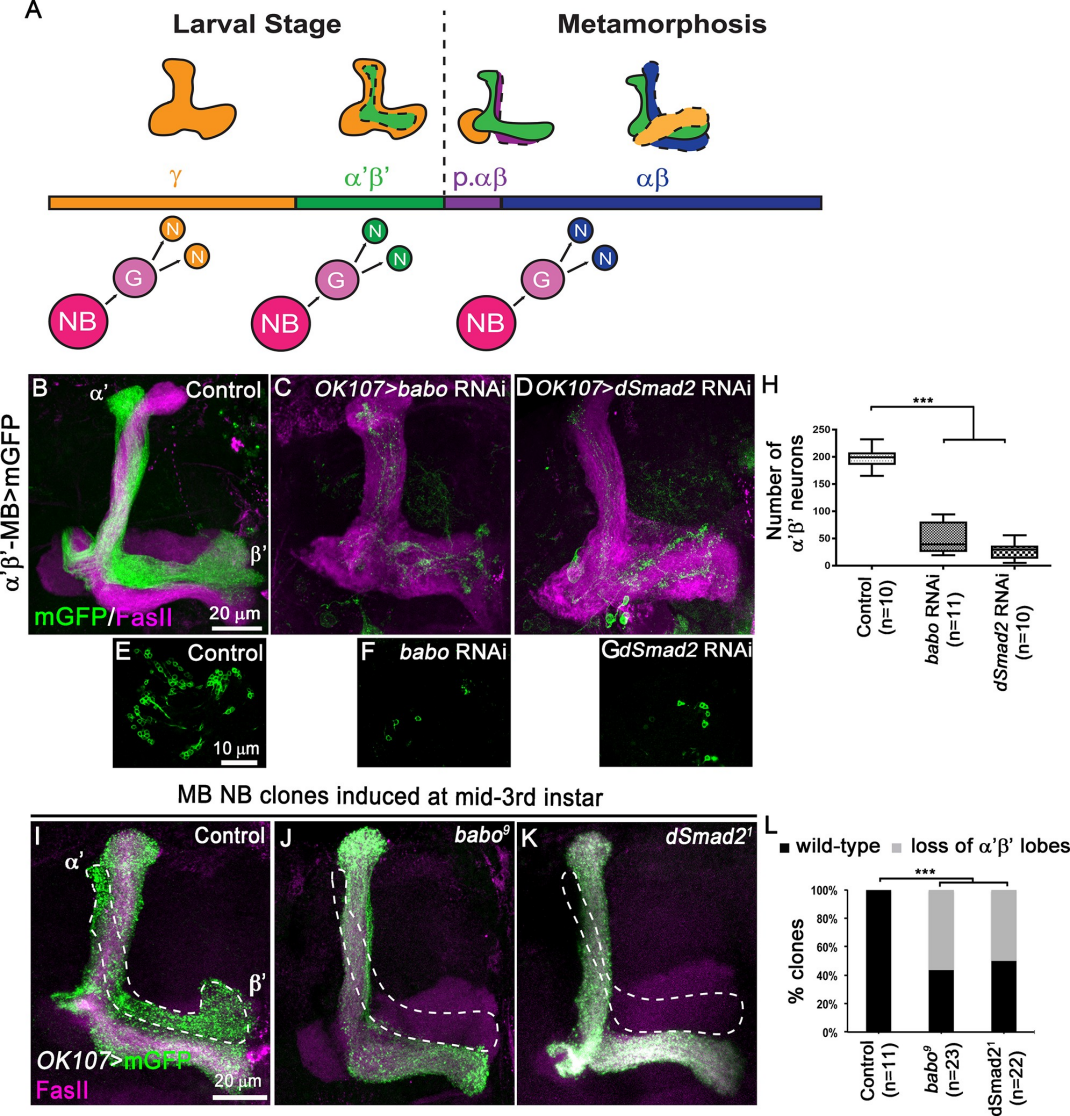
TGF-β signalling controls α’β’ MB neuron fate specification. (A) Schematic drawings representing the sequential generation of the four distinct subtypes of MB neurons, which can be classified based on their axonal projection patterns (orange, γ neurons; green, α’β’ neurons; purple, pioneer αβ neurons; blue, αβ neurons). MB neuroblasts (NBs) undergo multiple rounds of asymmetric divisions to generate ganglion mother cells (G) that in turn divide to produce two postmitotic neurons (N). (B-D) Adult MB lobes from control (B), *OK107>babo* RNAi (C) and *OK107>dSmad2* RNAi (D) brains stained with anti-FasII antibody (magenta). (E-G) Single confocal section thought the cell body cluster of adult MB neurons from control (E), *OK107>babo* RNAi (F) and *OK107>dSmad2* (G) brains. Green: GMR26E01*-*LexA-α’β’*-*MB-driven mGFP (mCD8::GFP) in B-G. (H) Quantification of the number of adult α’β’ MB neuron cell bodies from control, *OK107>babo* RNAi and *OK107>dSmad2* RNAi brains. Statistical comparison to the control: ***, p<0.001 (two tailed *t* test). (I-K) Adult MB lobes from control (I), *babo*^*9*^ (J) and *dSmad2*^*1*^ (K) neuroblast MARCM clones generated at mid-third instar, labelled with mGFP (green) using the GAL4-*OK107* driver and stained with anti-FasII antibody (magenta). (L) Quantification of α’β’ MB fate defects in control, *babo*^*9*^ and *dSmad2*^*1*^ neuroblast clones. Statistical comparison to the control: ***, p<0.001; (Fisher’s exact test).

One of the lineages governed by both intrinsic programs and extrinsic cues is represented by the *Drosophila* mushroom body (MB) neurons. The MBs are symmetric neuropiles essential for learning and memory [7]. They derive from four type I neuroblasts (NBs) that divide asymmetrically to produce ganglion mother cells (Gs) which in turn generate two postmitotic neurons [2] (Fig 1A). The four major types of MB neurons (γ, α’β’, pioneer αβ and αβ) are sequentially produced during development [8]. Early-born γ and α’β’ neurons arise during embryonic and early larval stages, or during late larval stages, respectively. Late-born pioneer αβ (p. αβ) and finally αβ neurons are generated during early metamorphosis [8] (Fig 1A). These functionally distinct types of MB neurons display characteristic adult axonal projections: αβ, p. αβ and α’β’ neuron axons bifurcate to produce dorsal and medial branches, while the larval medial and dorsal axonal branches of γ neurons are pruned during early metamorphosis and replaced by a single adult branch extending medially [8]. The generation of these different neuronal fates is dependent on the steroid hormone ecdysone, which in turn is tuned to the nutritional state of the animal [5,9]. The beginning of metamorphosis is initiated by an ecdysone peak arising during the prepupal stage [9]. We have previously shown that this prepupal ecdysone peak together with the induction of nuclear ecdysone receptor B1 (EcR-B1) expression promotes the transition from early-born to late-born MB neurons at the onset of metamorphosis [5]. However, we are just starting to understand the complex regulation of the response elicited by the timed ecdysone signal. In this study, we show that TGF-β signalling induced by the glial-derived Myoglianin (Myo) ligand is required in MB neuronal progenitors to consolidate the α’β’ fate by promoting the expression of EcR-B1 in postmitotic neurons. In contrast, the zinc finger transcription factor Kr-h1 antagonises the effect of TGF-β signalling, thus modulating the ecdysone signalling response to maintain the correct neuronal identity. Our data support the view that when this consolidation process is disrupted, α’β’ neurons lose their fate and convert to p.αβ neurons.

## Results

### Blocking TGF-β signalling reduces the number of early-born α’β’ MB neurons

During brain development, MB NBs sequentially generate the four main subtypes of MB neurons (Fig 1A). Previous work described the TGF-β/Activin type I receptor Baboon (Babo) and its intracellular effector dSmad2 as positive regulators of axonal remodelling in γ neurons. Both components of the TGF-β signalling cascade are required for the expression of the EcR-B1 isoform and the consequent activation of ecdysone signalling in MB neurons [10]. This positive regulation of ecdysone signalling led us to hypothesize that TGF-β signalling might play a role also in the definition of MB neuron temporal identity. To address this hypothesis we concentrated on α’β’ early-born MB neurons, which start to be generated during late larval stages, but do not undergo major neuronal remodelling during metamorphosis [8]. We knocked-down Babo or dSmad2 in all MB neurons using the GAL4-*OK107* driver. Simultaneously, we utilized the LexA binary system [11] to independently visualize the α’β’ MB subpopulation (Fig 1B–1D). We additionally immuno-labelled endogenous Fasciclin II (FasII), which in the adult MB strongly highlights the αβ lobes and weakly the γ lobe. Remarkably, an abnormal low number of α’β’ axons was observed upon inactivation of *babo* or *dSmad2* in MB neurons (Fig 1C and 1D), which corresponded to a dramatic decrease in the number of α’β’ neurons (Fig 1E–1H). However, the total number of MB cells at adult stage appeared to be unaffected upon inactivation of *babo* (S1A and S1B Fig). Moreover, preventing cell death by expressing the repressor p35 [12] did not restore the production of α’β’ neurons in GAL4-*OK107*-driven *dSmad2* RNAi brains (S1C–S1E Fig). To confirm the observed defects in α’β’ neuron specification using independent methods, we generated MB MARCM (mosaic analysis with a repressible cell marker [13]) neuroblast clones mutant for *babo* or d*Smad2* at mid-3rd instar (3,5 days (d) after larval hatching (ALH)), targeting specifically the α’β’ and αβ MB neuron populations (Fig 1I–1K). Compared to control (Fig 1I and 1L), *babo* or d*Smad2* mutant neuroblast clones showed a loss of α’β’ axonal lobes (Fig 1J–1L, dotted lines). Notably, the αβ axonal lobes were not affected in *babo* and *dSmad2* MARCM clones, suggesting that TGF-β signalling is not required for the specification of these late-born cell types. Overall, these data suggest that TGF-β signalling is required during MB development for the specification of adult α’β’ MB neurons.

### TGF-β signalling is dispensable for the initial generation and specification of α’β’ MB neurons

**Fig 2:**
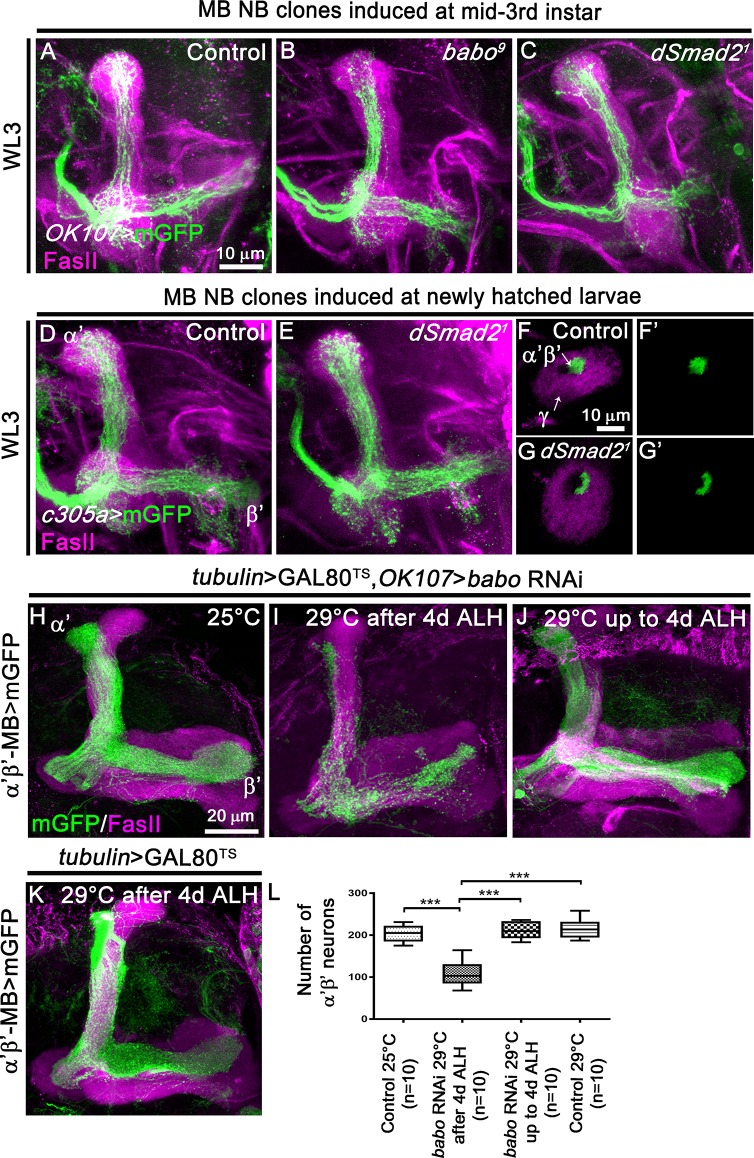
TGF-β signalling is dispensable for the initial α’β’ MB neuron production. (A-C) WL3 MB lobes from control (A) (n = 8), *babo*^*9*^ (B) (n- = 7) and *dSmad2*^*1*^ (C) (n = 12) MARCM NB clones induced at mid-third instar stage, labelled with mGFP (green) using the GAL4-*OK107* driver and stained with anti-FasII antibody (magenta). (D, E) WL3 MB lobes from control (D) (n = 5) and *dSmad2*^*1*^ (E) (n = 9) MARCM NB clones generated in newly hatched larvae, labelled with mGFP (green) using the α’β’-specific GAL4-*c305a* driver and stained with anti-FasII antibody (magenta). (F-G’) Cross sections of control (F,F’) and *dSmad2*^*1*^ (G,G’) WL3 MB peduncles from neuroblast MARCM clones generated in newly hatched larvae labelled with mGFP (green) using the *GAL4-c305a* driver and stained anti-FasII antibody (magenta). The arrows indicate the γ axons (FasII-positive area) in the outer layer and the α’β’ axons in the core layer (FasII-negative area). (H, I) Adult MB lobes from *tubulin>GAL80*^*TS*^,*OK107>babo* RNAi animals raised at 25°C (H) or at 29°C starting from 4d ALH (I). (J) Adult MB lobes from *tubulin>GAL80*^*TS*^,*OK107>babo* RNAi animals raised at 29°C and switched to 25°C starting from 4d ALH. (K) Adult MB lobes from *tubulin>GAL80*^*TS*^ RNAi animals raised at 29°C starting from 4d ALH. The temperature shift is at 4d after larval hatching (ALH) corresponding to the late larval stage. Magenta: anti-FasII staining. Green: GMR26E01*-*LexA-α’β’*-*MB-driven mGFP in F,I. (L) Quantification of number of adult α’β’ MB neuron cell bodies from the temperature-dependent *babo* RNAi experiments in H-K. Statistical comparison to the control: ***, p<0.001 (two tailed *t* test).

α’β’ neurons are produced starting from 3d ALH until 6 hours before puparium formation (BPF) [8]. Surprisingly, *babo* or d*Smad2* mutant MB neuroblast clones induced via MARCM in newly hatched larvae (NHL) and observed at the late wandering third-instar larval stage (WL3) did not show any obvious morphological defects compared to control neuroblast clones (S2A–S2C Fig). Similar results were obtained in knockdown experiments (S2D–S2I’ Fig). To discriminate between the production of γ and α’β’ neurons, we induced *babo* or d*Smad2* mutant MB MARCM neuroblast clones at mid-third-instar larval stage and analysed the clones at late WL3. Interestingly, in absence of TGF-β signalling we still observed the presence of axonal projections suggesting that MB neuroblasts continued to generate neurons during the developmental time window associated with α’β’ production (Fig 2A–2C). To test if these neurons are initially specified as α’β’, we induced neuroblast clones in newly hatched larva and visualised the clones with the GAL4-*c305a* driver which is mainly expressed in α’β’ neurons at WL3 [5]. We observed that blocking TGF-β signalling did not alter the initial specification of α’β’ fate (Fig 2D–2G’). Overall, these data support the view that TGF-β signalling is dispensable for the initial production and specification of α’β’ neurons. To support these findings, we performed a temperature-shift experiment using the TARGET system to temporally control the expression of the UAS-*babo* RNAi transgene [14]. Such a system is based on a temperature-sensitive GAL80 (GAL80^TS^) protein which represses the transcriptional activity of GAL4. Therefore, by switching animals from permissive (25°C) to restrictive temperature (29°C), we were able to control the timing of RNAi expression. We knocked-down *babo* expression in all MB neurons from 4d ALH onwards and independently visualised the α’β’ MB subpopulation using the LexA binary system in control adults (Fig 2H and 2K) or after Gal4/UAS-driven RNAi mediated *babo* knock-down (Fig 2I and 2J). Targeted *babo* knockdown starting from 4d ALH and thus approximately 24 hours after α’β’ neurons start to be produced, led to a significant reduction of α’β’ MB neurons (Fig 2I and 2L). Conversely, restricting the RNAi against *babo* until 4d ALH did not affect α’β’ MB production (Fig 2J and 2L). Taken together, these data suggested that while TGF-β signalling appears to be dispensable for the production of α’β’ neurons before metamorphosis, it is required to consolidate and maintain the α’β’ fate to adult life.

### TGF-β signalling promotes α’β’ MB neuron specification by inducing EcR-B1 expression

The intrinsic fate determinants Chronologically inappropriate morphogenesis (Chinmo) and Abrupt (Ab) have been implicated in MB neuron fate determination governing the fate switch between early-born versus late-born MB neurons [15, 16]. However, analysis of MB NB clones induced at NLH and observed at late WL3 revealed no obvious changes in Chinmo or Ab protein levels when TGF-β signalling was blocked suggesting that TGF-β signalling is dispensable for Chinmo or Ab expression in MB neurons (S3 Fig). Moreover, constitutive activation of the TGF-β pathway did not rescue the *chinmo*^*1*^ mutant fate defects in developing α’β’ neurons (S4A–S4E Fig). Loss of Chinmo abolishes EcR-B1 expression at WL3 [5]. Notably, EcR-B1 expression in *chinmo*^*1*^ mutant MB neurons was not restored upon activation of the TGF-β pathway (S4F–S4I’ Fig). Altogether these data suggest that Chinmo and TGF-β signalling are concurrently required to promote the expression of EcR-B1.

**Fig 3:**
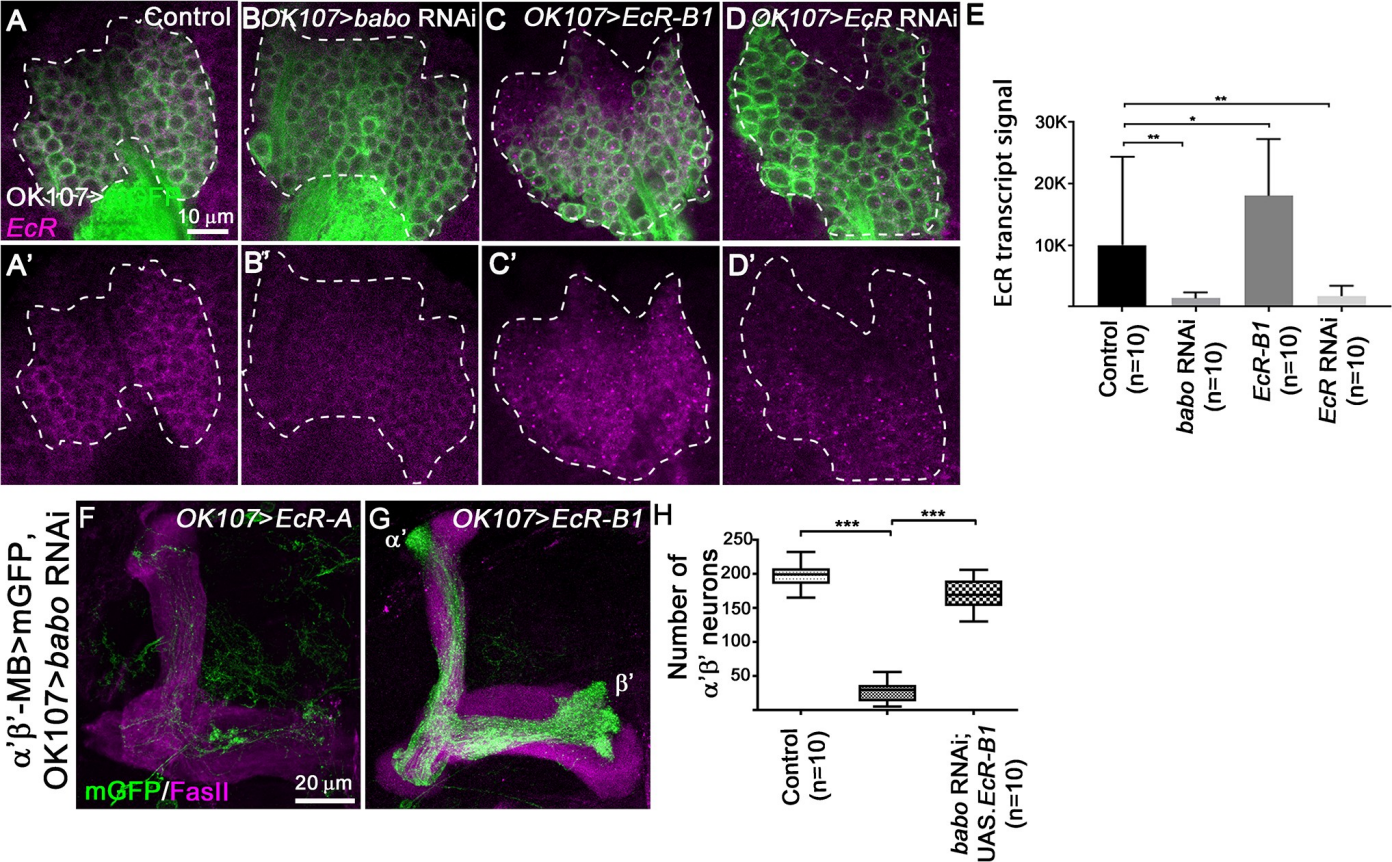
EcR-B1 isoform mediates TGF-β signalling-dependent α’β’ MB neuron specification. (A-D’) WL3 MB cell bodies from control (A), *OK107>babo* RNAi (B), *OK107>EcR-B1* (C) and *OK107>EcR* RNAi (D) brains stained with anti-*EcR* RNA probe (magenta). Green: GAL4-*OK107*-driven mGFP (A-D). (E) Quantification of *EcR* signal in control, *OK107>babo* RNAi, *OK107>EcR-B1* and *OK107>EcR* RNAi brains. 10 brains were analysed for each condition. Statistical comparison to the control: *, p<0.05; **, p<0.01 (Mann-Whitney test). (F,G) Adult MB lobes from *OK107>babo* RNAi, *EcR-A* (F) and *OK107>babo* RNAi, *EcR-B1* (G) brains stained with anti-FasII antibody (magenta). Green: GMR26E01*-*LexA-α’β’*-*MB-driven *mGFP* in F,G. (H) Quantification of number of adult α’β’ MB neuron cell bodies from control, *OK107>babo* RNAi, *EcR-A* and *OK107>babo* RNAi, *EcR-B1* brains. Statistical comparison to the control or to *OK107>babo* RNAi, *EcR-A* conditions: ***, p<0.001 (two tailed *t* test).

Previous work proposed that TGF-β signalling promotes the transcription of the EcR-B1 gene in MB neurons at the late larval stage [10]. To confirm this hypothesis we performed RNA FISH (Florescent *In Situ* Hybridization) to visualise *EcR* transcripts [17]. We detected a positive signal for the *EcR* probe in late WL3 control MB neurons (Fig 3A and 3E). This signal was significantly enhanced in MB neurons overexpressing *EcR* (Fig 3C and 3E), but reduced when the expression of *EcR* was knocked down (Fig 3D and 3E) supporting the specificity of the *EcR* probe. Interestingly, knockdown of *babo* led to a strong reduction of the *EcR* probe signal (Fig 3B and 3E), suggesting that TGF-β signalling positively regulates EcR-B1 transcription in MB neurons.

To determine whether TGF-β signalling promotes MB fate consolidation by specifically upregulating EcR-B1, we expressed EcR-A or EcR-B1 isoforms together with *babo* RNAi inducing constructs using the GAL4-*OK107* driver. We independently visualized the α’β’ MB subpopulation with a LexA driver. While EcR-A isoform expression did not rescue GAL4-*OK107*-driven *babo* RNAi phenotypes (Fig 3F and 3H), expressing the EcR-B1 isoform significantly restored the number of α’β’ neurons (Fig 3G and 3H). Thus, the TGF-β pathway consolidates α’β’ neuron identity by promoting the expression of the EcR-B1 receptor.

### TGF-β signalling is required in progenitor MB neurons for α’β’ MB neuronal fate

**Fig 4:**
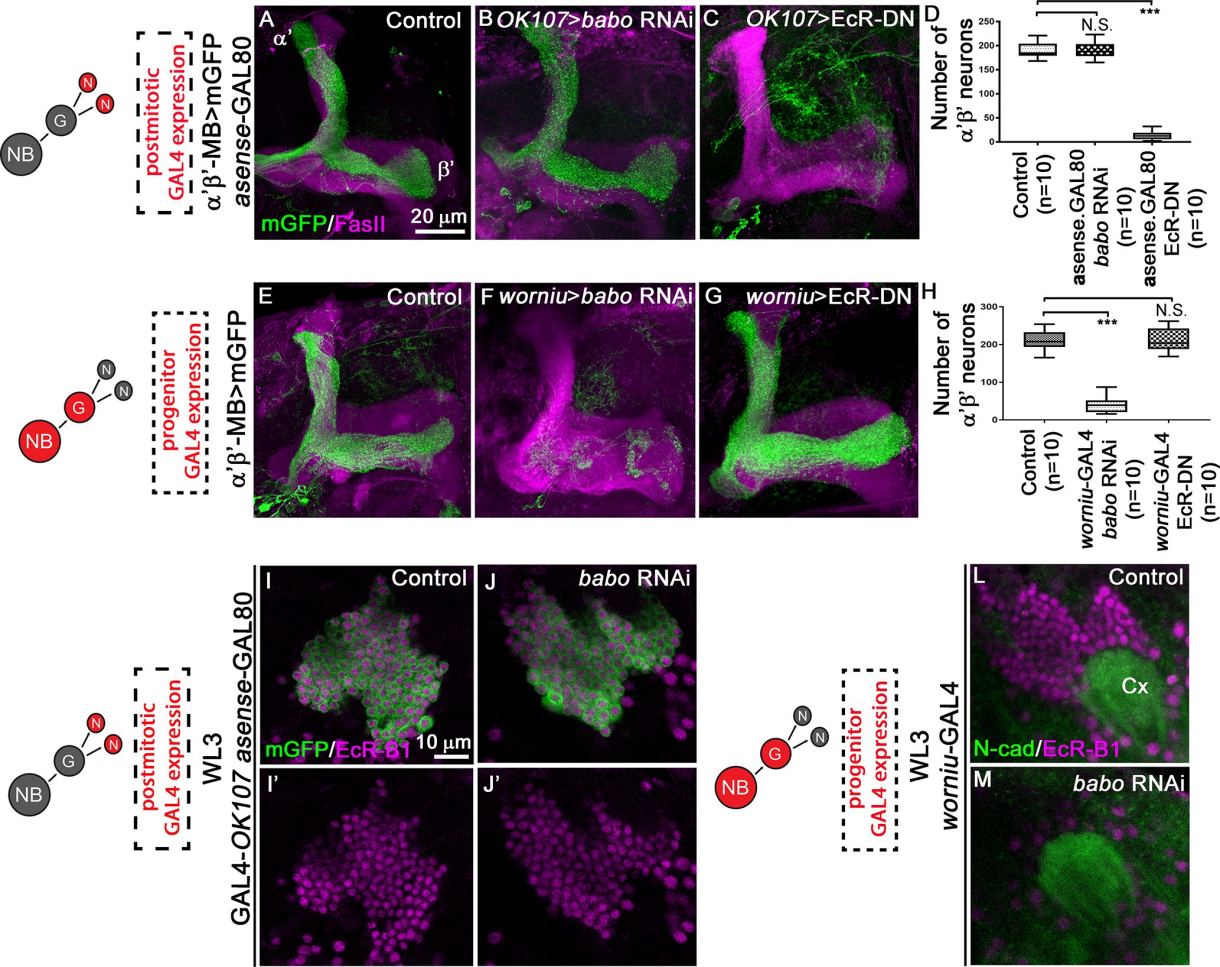
TGF-β signalling is required in MB progenitors to control EcR-B1 expression during consolidation of α’β’ MB fate. (A-C) Adult MB lobes from control (A), *OK107>babo* RNAi *asense*>GAL80 (B), and *OK107>*EcR-DN *asense*>GAL80 (C) brains stained with anti-FasII antibody (magenta). Green: GMR26E01*-*LexA-α’β’*-*MB-driven mGFP in A-C. (D) Quantification of the number of adult α’β’ MB neuron cell bodies from control, *OK107>babo* RNAi *asense*>GAL80 and *OK107>*EcR-DN *asense*>GAL80 brains. Statistical comparison to the control: N.S. Not Significant; ***, p<0.001 (two tailed *t* test). (E-G) Adult MB lobes from control (E), *worniu>babo* RNAi (F), and *worniu>*EcR-DN (G) brains stained with anti-FasII antibody (magenta). Green: GMR26E01*-*LexA-α’β’*-*MB-driven mGFP in E-G. (H) Quantification of number of adult α’β’ MB neuron cell bodies from control, *worniu>babo* RNAi and *worniu>*EcR-DN brains. Statistical comparison to the control: N.S. Not Significant; ***, p<0.001 (two tailed *t* test). (I-J’) WL3 MB cell bodies from control (I,I’) and *OK107>babo* RNAi asense>GAL80 (J, J’) brains stained with anti-EcR-B1 antibody (magenta). Green: GAL4-*OK107*-driven *mGFP* in I-J’. (L-M) WL3 MB cell bodies from control (L) and *worniu>babo* RNAi (M) brains stained with anti-EcR-B1 antibody (magenta). Anti-N-cadherin (green) staining was used as landmark in locating MB neurons. Cx, MB Calyx. NB, neuroblast; G, ganglion mother cell; N, neurons.

One possible explanation for the observed TGF-β signalling-dependent decrease in α’β’ neurons is that TGF-β signalling acts in MB progenitors to maintain α’β’ identity. To specifically manipulate mature neurons, we used *asense*-GAL80 that suppresses the activity of *OK107*-driven GAL4 in MB NBs and Gs [18]. Strikingly, while the knockdown of *babo* exclusively in post-mitotic MB neurons did not lead to fate defects (Fig 4A, 4B and 4D), blocking ecdysone signalling postmitotically was sufficient to inhibit α’β’ neuron fate (Fig 4A, 4C and 4D). Conversely, when we restricted the knockdown of *babo* to the NBs using *worniu*-GAL4 [12], the production of α’β’ MB neurons was severely reduced (Fig 4E, 4F and 4H). In contrast, disruption of ecdysone signalling selectively in NBs did not cause any significant decrease in α’β’ MB neuron number (Fig 4E, 4G and 4H). These data indicate that the generation of the different MB subpopulation fates requires a functional TGF-β pathway in neuronal progenitors and the activation of ecdysone signalling in postmitotic neurons. To better refine the temporal expression pattern of EcR-B1, we examined its distribution in late WL3 MB neurons upon inactivation of TGF-β signalling either in mature neurons or in progenitors. EcR-B1 expression in late WL3 MB neurons is mostly restricted to *worniu*-negative postmitotic neurons (S5 Fig) [19]. When TGF-β signalling was impaired only postmitotically, EcR-B1 expression was not altered (Fig 4I–4J’). However, in accordance with the instructive role of TGF-β signalling in MB precursors, knocking-down *babo* only in MB progenitors led to suppression of EcR-B1 expression (Fig 4L and 4M). Moreover, expressing the EcR-B1 in MB progenitors did not rescue the production of α’β’ MB neurons upon knockdown of *babo* (S6 Fig). Overall, these data indicate that TGF-β pathway and ecdysone signalling act sequentially at distinct stages to control neuronal fate. We propose that this sequence promotes the maintenance of α’β’ MB fate.

### Glial Myoglianin activates TGF-β signalling during α’β’ MB neuronal fate determination

**Fig 5:**
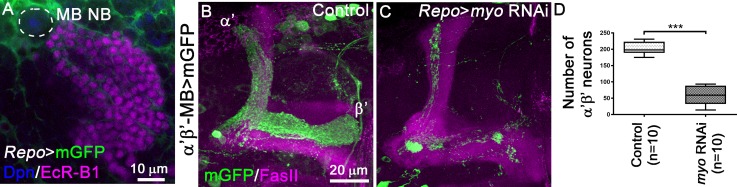
Reduced levels of Myoglianin in the glia lead to reduction of α’β’ MB neuron number. (A) MB neuroblast (Nb) stained with anti-Dpn antibody (blue), MB neurons stained with anti-EcR-B1 antibody (magenta) used as landmark in locating MB neuroblast and glia labelled with *GAL4-Repo*-driven mGFP (green). (B,C) Adult MB lobes from control (B) and *Repo>myo* RNAi (C) brains stained with anti-FasII antibody (magenta). Green: GMR26E01*-*LexA-α’β’*-*MB-driven mGFP in B,C. (D) Quantification of number of adult α’β’ MB neuron cell bodies from control and *Repo>myo* RNAi brains. Statistical comparison to the control: ***, p<0.001 (two tailed *t* test).

Among the seven TGF-β reported ligands, Myo is the ligand for the Babo receptor in MB neurons that enables the expression of EcR-B1 at late larval stage [20]. Myo is secreted by the larval cortex glia and the astrocyte-like glia cells [20]. We found that Repo-positive glia surrounds the cell bodies of MB NB at late WL3 (Fig 5A). Therefore, we tested whether the release of Myo from the glia compartment could be essential for MB fate transition. We knocked-down Myo in glial cells using the *Repo*-GAL4 driver and simultaneously visualized the α’β’ MB subpopulation with a LexA driver. Expression of *myo* knock-down constructs in Repo-positive glia cells led to a strong reduction of both axons and cell number of α’β’ MB neurons (Fig 5B–5D). These results suggested that glia cells-derived Myo is necessary to mediate TGF-β-dependent control of α’β’ MB neuron fate.

### Impairment of TGF-β signalling leads to increased numbers of pioneer αβ MB neurons

**Fig 6:**
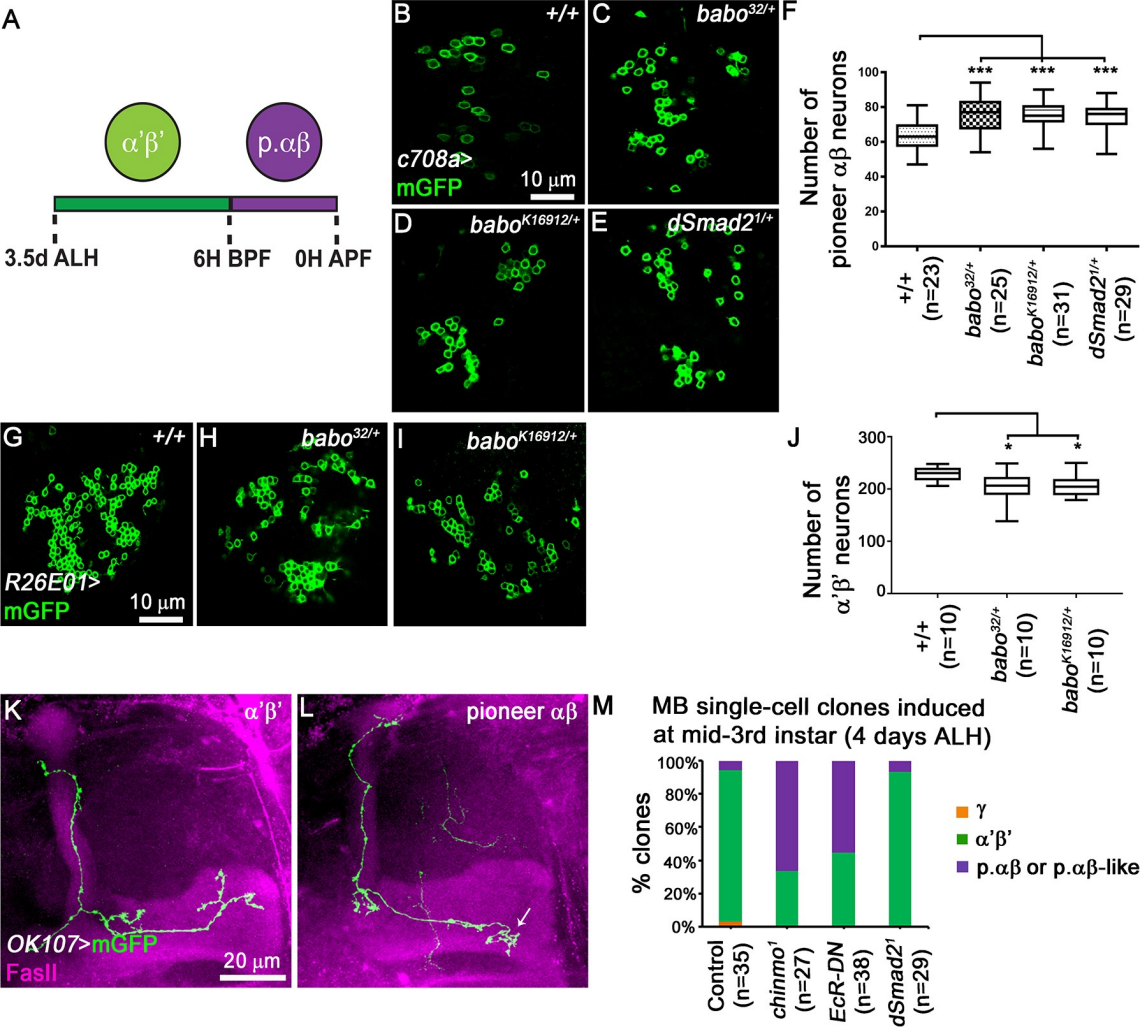
The numbers of pioneer αβ MB neurons is affected upon TGF-β signalling impairment. (A) Schematic presentation of sequential production of α’β’ and p. αβ. ALH, after larval hatching; BPF, before puparium formation; APF, after puparium formation. (B-E) Single confocal section thought the cell body cluster of adult MB neurons from control (B), *babo*^*32/+*^ (C), *babo*^*K16912/+*^ (D) and *dSmad2*^*1/+*^ (E) brains. Green: c708a-driven mGFP in B-E highlighting p. αβ neurons. (F) Quantitative analysis of MB p. αβ neuron numbers after removing one copy of *babo* and/or *dSmad2* gene. MB p. αβ neurons were labelled by GAL4*-c708a*. Statistical comparison to the control: ***, p<0.001 (two tailed *t* test). (G-I) Single confocal section thought the cell body cluster of adult MB neurons from control (G), *babo*^*32/+*^ (H) and *babo*^*K16912/+*^ (I) brains. Green: GMR26E01*-*LexA-α’β’*-*MB-driven mGFP in G-I highlighting α’β’ neurons. (J) Quantitative analysis of the number of MB α’β’ neurons after removing one copy of the *babo* gene. MB α’β’ neurons were labelled by GMR26E01*-*LexA*-R26E01*. Statistical comparison to the control: *, p<0.001 (two tailed *t* test). (K, L) Adult axonal projections of single cell MARCM clone of α’β’ (K) and p. αβ (L) neurons. Arrow indicates the typical short medial axonal process of the p. αβ neurons [15]. (M) Percentages of different subtypes of MB neurons among control, *chinmo*^*1*^, *OK107*>EcR-DN and *dSmad2*^*1*^ single cell clones that were induced at mid-third instar stage. *chinmo*^*1*^ clones were used as positive control for the identity switch phenotype [15].

The p. αβ neurons are generated after α’β’ neurons, from 6 hours (h) before puparium formation (BPF) to 0h after puparium formation (APF) [15] (Fig 6A). Therefore, disruption of the final α’β’ fate might result in a fate-shift towards the p. αβ neurons. Thus, we tested whether the loss of α’β’ neurons might impact the p. αβ population. Due to the absence of specific LexA drivers to visualise the p. αβ neurons in GAL4-*OK107*-driven *babo* RNAi brains, we reduced the gene dosage of *babo* or *dSmad2* and used the GAL4-*c708a* driver to label specifically p. αβ neurons [15]. Removing one copy of *babo* or *dSmad2* led to an increase in the number of p. αβ neurons compared to wild-type animals (Fig 6B–6F). Conversely, decreasing *babo* gene dosage led to a reduction in the number of α’β’ neurons (Fig 6G–6J). These data suggest that reduced TGF-β signalling destabilizes α’β’ neuron identity and that possibly those neurons convert into p. αβ neurons. To support these data, we performed single-cell clonal analysis of post-mitotic neurons generated at mid third instar larvae. In control adult brains, the majority of neurons showed typical α’β’ axonal projections (Fig 6K and 6M). In contrast, 60% of neurons expressing a dominant negative form of EcR (EcR-DN) appeared to branch their axons as p. αβ (Fig 6L and 6M). In accordance with the instructive role of TGF-β signalling in MB precursors, single-cell clones for the d*Smad2*^*1*^ allele did not show any fate defects (Fig 6M). Overall these results suggest that, in absence of TGF-β signalling and consequently of ecdysone signalling, α’β’ neurons lose their fate and partially convert to p. αβ neurons.

### The antimorphogenetic Kr-h1 factor coordinates ecdysone response during α’β’ MB neuron production

**Fig 7:**
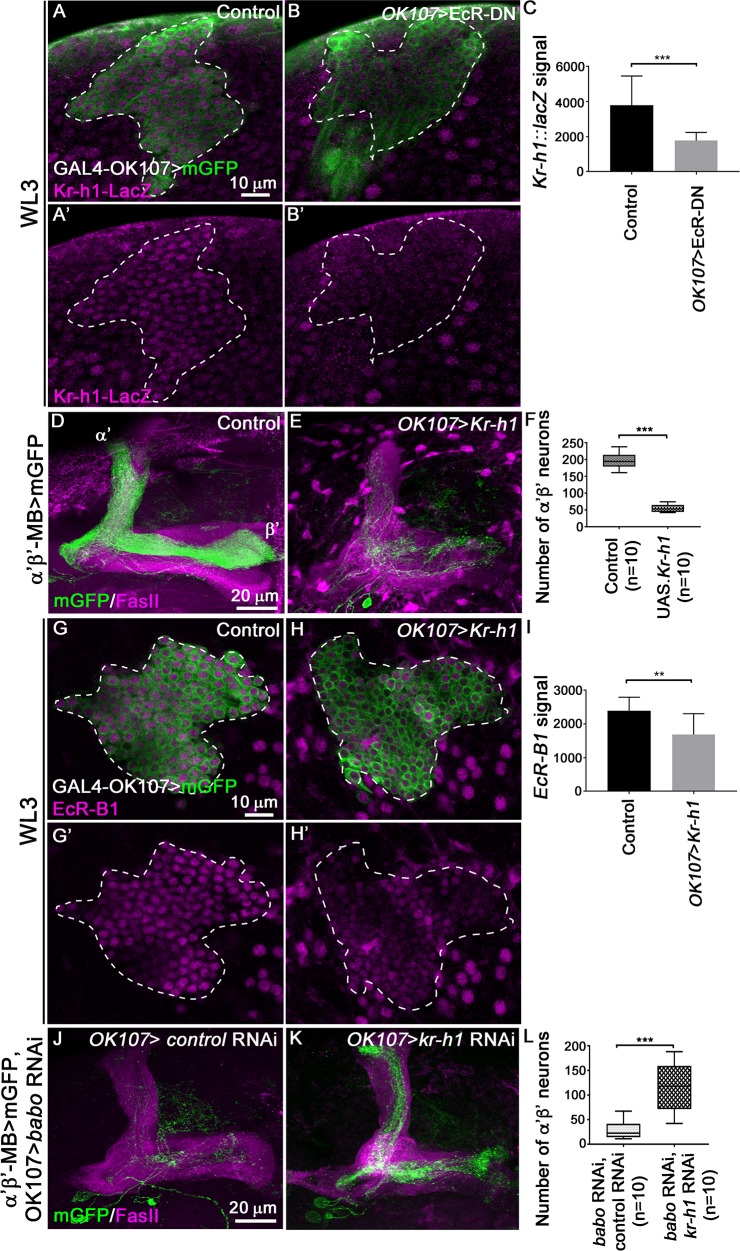
Kr-h1 acts downstream of ecdysone signalling to negatively regulate the specification of α’β’ MB neurons. (A-B’) WL3 MB cell bodies from control (A,A’) and *OK107>EcR-DN* (B, B’) brains carrying the *Kr-h1*^*10642*^::*LacZ* enhancer trap line revealed with anti-βGal antibody (magenta). Green: GAL4-*OK107*-driven mGFP in A-B’. (C) Quantification of *Kr-h1*^*10642*^::*LacZ* signal in control and *OK107>EcR-DN* brains. 10 brains were analysed for control or *OK107>EcR-DN* conditions. Statistical comparison to the control: ***, p<0.001 (Mann-Whitney test). (D-E) Adult MB lobes from control (D) and *OK107>Kr-h1* (E) brains stained with anti-FasII antibody (magenta). Green: GMR26E01*-*LexA-α’β’*-*MB-driven mGFP in D,E. (F) Quantification of the number of adult α’β’ MB neuron cell bodies from control and *OK107>Kr-h1* brains. Statistical comparison to the control: ***, p<0.001 (two tailed *t* test). (G-H’) WL3 MB cell bodies from control (G,G’) and *OK107>Kr-h1* (H, H’) stained with anti-EcR-B1 antibody (magenta). Green: GAL4*-OK107*-driven mGFP in G-H’. (I) Quantification of EcR-B1 signal in control and *OK107>Kr-h1* brains. 10 brains were analysed for both control and *OK107>Kr-h1* conditions. Statistical comparison to the control: **, p<0.01 (Mann-Whitney test). (J-K) Adult MB lobes from *OK107>babo* RNAi, *luciferase* RNAi (J) and *OK107>babo* RNAi, *Kr-h1* RNAi (K) brains stained with anti-FasII antibody (magenta). Green: GMR26E01*-*LexA-α’β’*-*MB-driven mGFP in J,K. (L) Quantification of number of adult α’β’ MB neuron cell bodies from *OK107>babo* RNAi, *luciferase* RNAi and *OK107>babo* RNAi, *Kr-h1* RNAi brains. Statistical comparison to the control: ***, p<0.001 (two tailed *t* test).

We next investigated whether the ecdysone response involved in MB fate consolidation was modulated by the presence of an extra level of regulation which might counteract the TGF-β signalling. We focussed our analysis on the zinc finger transcription factor Kruppel-homolog 1 (Kr-h1) based on several lines of evidence. Kr-h1 is a global regulator of prepupal ecdysone response [21, 22]. Although it shows a broad ecdysone-dependent expression in developing MB neurons, *Kr-h1* mutant MBs did not show obvious changes in neuronal morphology [23, 24]. However, in a different set of neurons, the DC neurons, reducing the levels of *Kr-h1* ameliorated TGF-β signalling-dependent morphological defects, suggesting that in this context physiological expression of Kr-h1 antagonizes the TGF-β pathway [23]. To test whether Kr-h1 plays a role in MB fate transition, we first confirmed the expression of Kr-h1 in MB neurons at late WL3 using a LacZ enhancer trap line that reports Kr-h1 expression [25]. In control brains (Fig 7A and 7C) Kr-h1-LacZ signal was detected in all MB neurons, whereas upon blocking ecdysone pathway the Kr-h1-LacZ signal was drastically reduced (Fig 7B and 7C). Hence, Kr-h1 expression in developing MB neurons appears to be positively regulated by the activation of ecdysone signalling. Interestingly, overexpression of Kr-h1 in developing MB neurons affects axonal MB morphology [23]. Therefore, we examined whether overexpression of Kr-h1 could also affect α’β’ MB neuron production. We overexpressed Kr-h1 using the GAL4-*OK107* driver and visualised the α’β’ MB subpopulation using a specific LexA driver. Increased levels of Kr-h1 induced loss of α’β’ axonal projections and a significant reduction in the number of α’β’ MB neurons (Fig 7D–7F). This reduction in α’β’ MB neurons was associated with decreased levels of EcR-B1 signal in late WL3 brains in which Kr-h1 was overexpressed using the GAL4-*OK107* driver (Fig 7G–7I). These data suggested that Kr-h1 negatively modulates the ecdysone response in MB neurons at the end of the larval stage. If Kr-h1 negatively influences the generation of α’β’ MB neurons, we expect that reduced levels of Kr-h1 would suppress the fate defects occurring in the absence of TGF-β signalling. Therefore, we knocked-down both *Kr-h1* and *babo* using GAL4-*OK107* and independently visualised α’β’ MB neurons using a specific LexA driver. The expression of control RNAi constructs together with *babo* RNAi under the control of GAL4-*OK107* resulted in a clear reduction of α’β’ MB neurons (Fig 7J and 7L) comparable to *babo* RNAi alone (see Fig 1C and 1H). In contrast, simultaneous knockdown of *Kr-h1* together with *babo* RNAi partially rescued the number of α’β’ MB neurons (Fig 7K and 7L). Altogether, these results suggest that Kr-h1 negatively regulates ecdsyone-dependent response in MB neurons.

**Fig 8:**
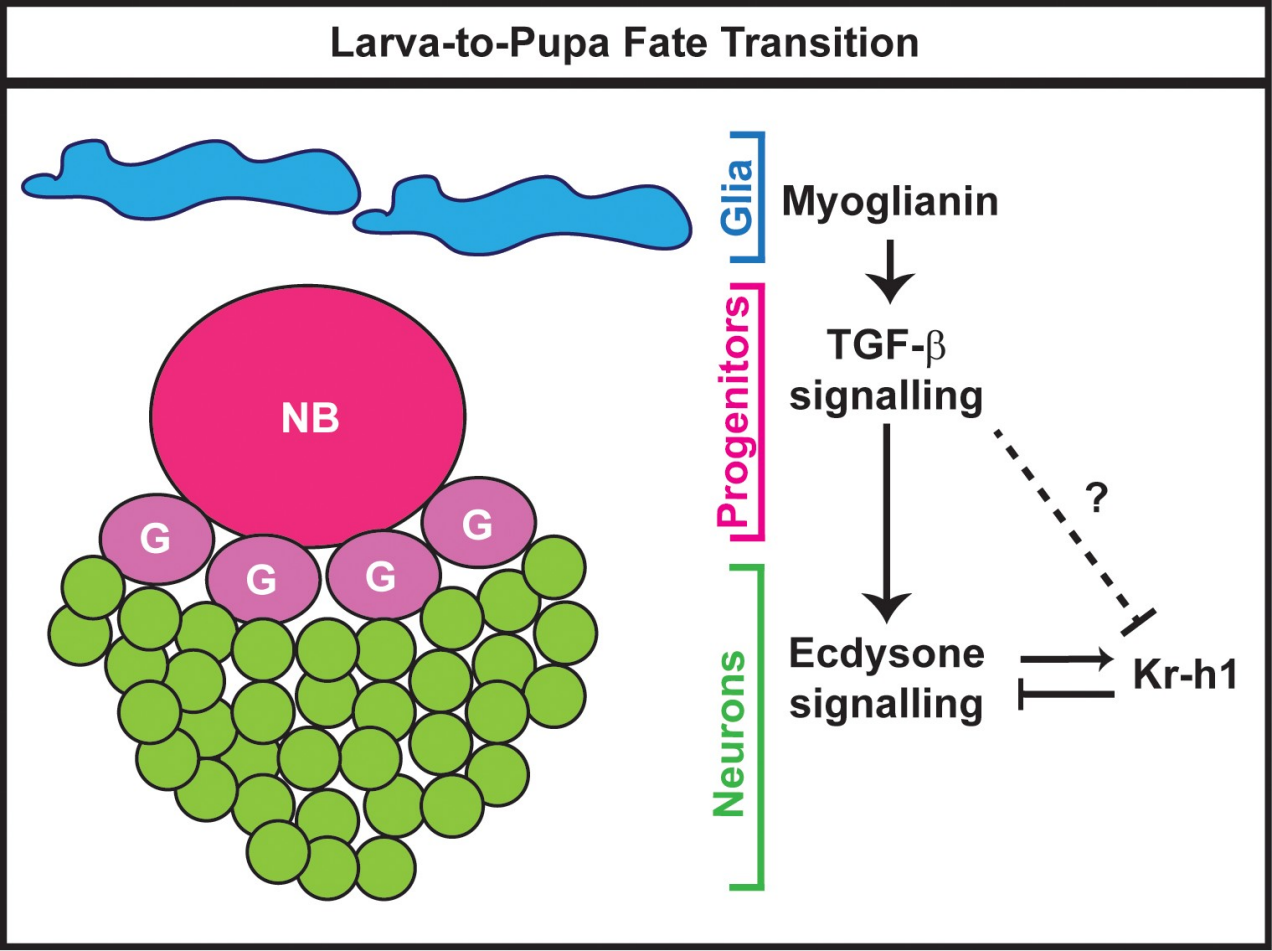
Model of ecdysone signalling modulation during the transition from α’β’ to p. αβ MB fate. The fate switch between early-born MB neurons (γ and α’β’) versus late-born MB neurons (p. αβ and αβ) occurs during the developmental transition from larval stage to metamorphosis. During this developmental time window, the Myoglianin ligand released from glial cells activates the TGF-β signalling in MB progenitors. As a result, the postmitotic MB neurons (green) are now capable of sensing the circulating hormone ecdysone. In turns, ecdysone signalling regulates the expression of Kr-h1 which negatively tunes ecdsyone response. The question mark reflects the possibility of a direct regulation of Kr-h1 by TGF-β signalling. NB, neuroblast; G, ganglion mother cell.

Taken together our data support a model in which glial cells release the Myo ligand to activate TGF-β signalling in the MB progenitors. Consequently, TGF-β signalling in NBs consolidates the final identity of α’β’ neurons by promoting the expression of EcR-B1 in the progeny neurons. In due order, the intrinsic factor Kr-h1 tunes ecdysone signalling response during this ecdysone-dependent consolidation phase (Fig 8).

## Discussion

Here we revealed a fundamental role for Myo-mediated TGF-β signalling in regulating fate specification of MB neurons. This signalling is initiated in the neuronal progenitors and we propose that it is necessary to consolidate the identity of newly born neurons by enabling them to sense and integrate the ecdysone hormonal signal. As modulator of this consolidation fate program, the factor Kr-h1 negatively regulates ecdysone signalling response and antagonises the TGF-β pathway.

### TGF-β signalling in fate specification

Evidence derived from vertebrate models indicates that the temporal competence of neuronal precursors to generate different neuronal subtypes is governed by the combination of cell-intrinsic programs and extrinsic cues [3]. In contrast, fate determination in the *Drosophila* nervous system appeared to be mainly determined by intrinsic cascades [2]. Only recently, first reports started indicating that extrinsic factors can modulate fate decisions in the nervous system of the fly [5, 6]. Thus, fate decisions in the fly nervous system might follow principles that are more relatable to the ones utilised in vertebrate lineages than previously expected. Along these lines, our current data revealed a central role of TGF-β signalling in temporal fate specification during MB development. In the rodent hindbrain, midbrain and spinal cord, TGF-β signalling constrains the neural progenitor potency to promote fate transition from early to late born cell types, acting as a temporal switch signal regulating the expression of intrinsic identity factors in young progenitors [26]. These similarities suggest that TGF-β might represent an evolutionary conserved extrinsic signal modulating temporal fate specification.

Our present data suggest that TGF-β signalling links the temporal neuronal fate program to developmental progression. Re-examination of the EcR-B1 expression in *dSmad2*^*1*^ mutant MB clones at late larval stages revealed a 12 hours delay in the onset of EcR-B1 expression leading to inability of MB neurons to respond to the prepupal ecdysone peak [27]. Thus, TGF-β signalling might help to synchronize the production of distinct MB neuron subtypes coordinating diverse developmental programs. Accordingly, we found that the glial Myo ligand mediates the TGF-β-dependent MB fate transition. Given that the prepupal ecdsyone peak is triggered after the larva reaches the critical weight point [9], we hypothesise that glia serve as nutrition sensors in the brain during larval development and could be coordinating developmental timing of the fate specification program.

### Fate consolidation

Although α’β’ neurons are born during the larval stage, based on their immature dendrites and axons, and on the absence of functional response in appetitive olfactory learning behaviour, it appears that they are not fully differentiated at the end of larval life [8, 28]. Therefore, the initial state of these immature α’β’ neurons could be labile. Their immature neurite trajectories might possess a certain degree of morphological plasticity, since at early pupal stages the axonal lobes are primarily made of α’β’ axons, after γ axons have completely pruned. Indeed, our data provide strong support for the presence of an active consolidation signal required to maintain α’β’ fate at adult stage. In fact, after impairment of TGF-β signalling, neurons born in the time window corresponding to the production phase of α’β’ displayed the expected axonal pattern for α’β’ neurons and expressed an α’β’ marker before metamorphosis. Taken these data together, the alternative hypothesis that TGF-β signalling could be involved in the initial specification of α’β’ MB neurons at mid-third instar appears much more unlikely. Notably, studies on fate specification in vertebrate systems have described a postmitotic fate consolidation event for developing motor and cortical neurons [29, 30, 31]. In particular, the homeobox gene HB9 has an essential function in maintaining the fate of the motor neurons by actively suppressing the alternative V5 interneuron genetic program [29, 30]. Indeed, mice lacking HB9 function showed a normal number of motor neurons that acquired, though, molecular features of V5 interneurons [29, 30]. Interestingly, in absence of HB9 motor neurons are initially specified and they retain their characteristic axonal projection [29, 30]. Similarly, the expression of the retinoic acid receptor (RAR) is required to maintain the fate of layer V-III cortical neurons, and when the expression of RAR is abolished these neurons acquire the identity of layer II cortical neurons [31]. These similarities in fate consolidation programs might reflect a common strategy in both invertebrates and vertebrates to first specify and then refine neuronal fate, according to the appropriate context.

### A complex molecular network controls neural fate specification

Recently, RNA profiling analysis of MB neurons at different developmental time points uncovered a complex feedback regulation network that governs EcR expression [24]. This combination of positive as well as negative feedback loops is required to coordinate EcR expression levels and its temporal regulation during brain development [24]. Our FISH analysis suggested that TGF-β signalling promotes the transcription of the EcR-B1 gene in MB neurons at late wandering larval stage. Although detectable EcR-B1 protein is restricted to postmitotic MB neurons (S4 Fig) [19], our genetic data revealed that TGF-β signalling is necessary in the MB progenitors to allow the expression of EcR-B1. This evidence raises the possibility that TGF-β signalling promotes the transcription of *EcR* gene in neuronal progenitors and potentially post-transcriptional mechanisms are involved to narrow down the translation of the EcR-B1 receptor only postmitotically. However, our data are against this hypothesis, since expression of EcR-B1 specifically in MB progenitors did not rescue the TGF-β signalling-dependent fate defects. Moreover, given that TGF-β signalling is required to consolidate the fate of the larval-born α’β’ neurons at the end of larval stage, suggests that the TGF-β pathway regulates a consolidation fate process independently of cell division. In this scenario, the expression of EcR-B1 in the newly born neurons could be promoted via a cell-to-cell communication signalling cascade initiated in neuronal progenitors by the activity of TGF-β signalling. Examples of this type of signal transmission are represented by the juxtacrine signalling mediated by Notch, Semaphorin or Ephrin pathways [32]. In particular, the intercellular interaction between Notch and its ligand Delta in neighbouring cells is fundamental to direct cell fate decisions [33].

In addition to an upstream regulation of ecdysone signalling, we uncovered the intrinsic factor Kr-h1 as a downstream modulator of the ecdysone-dependent fate consolidation program. Interestingly, the transition from larval stage to metamorphosis is regulated by the balance of the two major hormones, the juvenile hormone (JH) and ecdysone. JH prevents metamorphosis by the induction of the transcription factor Kr-h1 within the ring gland, which in turn suppresses the up-regulation of the ecdysone-dependent metamorphic genes *E93* and *Broad Complex* [34]. The TGF-β/Activin pathway contributes to decreasing Kr-h1 expression via E93 allowing the beginning of metamorphosis [35]. Along these lines, the antagonism between ecdysone and JH through Kr-h1 could potentially regulate the MB temporal fate cascade at the onset of metamorphosis.

In conclusion, our work shed light on the intrinsic and extrinsic mechanisms regulating the consolidation of the terminal fate. Understanding these processes will help us gain insights into their dysregulation in neurodevelopmental disorders and into their role in stem cell reprogramming.

## Materials and methods

### Flies

Fly stocks used were as follows: *UAS-Kr-h1-HA* [FlyORF#F000495], *GAL80-asense* [18], *UAS-dSmad2-CA* [36] and *babo*^*9*^ [10]. The following stocks were obtained from the Bloomington Stock Center (BSC): *hs-FLP*, *tubP-GAL80*, *FRT19A*; *UAS-mCD8*::*GFP* (BL#5134), *tubP-GAL80*, *FRTG13* (BL#5140), *tubP-GAL80*, *FRT40A* (BL#5192), *hs-FLP*, *UAS-mCD8*::*GFP* (BL#28832), *UAS-mCD8*::*GFP* (BL#5130), *GAL4-c708* (BL#50743), *GAL4-Repo* (BL#7415), *UAS-Myoglianin* RNAi (BL#31200) [37, 38, 39], *UAS-EcR-B1*^*ΔC655*.*W650A*^ (BL#6872), *tub-GAL80*^*TS*^ (BL#7017), *UAS-EcR-B1* (BL#6469), *UAS-EcR-A* (BL#6470), *LexA-GMR26E01* (α’β’-driver; BL#54617), *LexAop-mCD8-GFP* (BL#32203; BL#32205), *UAS-mCD8-GFP* (BL#32194), *GAL4-OK107* (BL#854), *UAS-Luciferase* RNAi (BL#31603), *UAS-Kr-h1* RNAi (BL#50685), *UAS-LacZ* (BL#1777), *GAL4-worniu* (BL#56553), GAL4-*c305a* (BL#30829), d*Smad2*^*1*^ (BL#44384), *babo*^*32*^ (BL#5399), *babo*^*k16912*^ (BL#11207), *chinmo*^*1*^ (BL#59969), *UAS-babo-CA* (BL#64293) and *Kr-h1*^*10642*^ (BL#12380). The following stocks were obtained from Vienna *Drosophila* RNAi Centre (VDRC): *babo* RNAi (v106092), *dSmad2* RNAi (v14609) and *EcR* RNAi (v37059). A full list of used genotypes is included in the supplemental information. Mosaic analyses using MARCM to generate NB or single cell clones were performed as previously described [8, 13]. Collected eggs were incubated for 20 hours at 25°C. The newly hatched larvae or mid-third instar larvae (3,5 days ALH) were heat shocked at 37°C in a water bath for one hour and then returned to 25°C.

### Antibodies

Primary antibodies used in this study were: anti-Fasciclin II (DSHB, 1D4, 1:15), anti-EcR-B1 (DSHB, AD4.4, 1:10), anti-Trio (DSHB, 9.4A, 1:5), anti-GFP (Molecular Probes, 1:1000), anti-Deadpan (Abcam, 1:100), anti-β-Gal (MP Biomedicals, 1:500), anti-Chinmo ([40], 1:500), anti-Abrupt ([41], 1:50) and anti-DN-Cadherin (DSHB, DN-Ex, 1:50).

### Immunostaining, imaging and image analysis

Brains were dissected in cold PBS, fixed in 4% formaldehyde and incubated overnight at 4°C with primary antibody. Secondary antibody was incubated for four hours at room temperature. Brains were mounted in Vectashield mounting media (Vector Laboratories). Images were collected with a Zeiss LSM 780 Meta confocal microscope using a 40× 1.4 NA oil immersion objective. To count cell numbers, cell bodies were identified based on mGFP signal. In Fig 4, the analysis was restricted to 4–5 days old female animals. Fluorescence measurements were performed using ImageJ [42]. An outline was drawn around each area of interest and area, integrated density and mean grey value were measured, along with adjacent background readings. The corrected total cellular fluorescence (CTCF) = integrated density–(selected area × mean fluorescence of background readings), was calculated.

### RNA FISH protocol

Wandering third instar larva brains were dissected in cold PBS. Fixation, hybridization and immunostaining were performed as previously described [17]. A set of 30 oligonucleotide probes specific to *EcR* coding sequence was created using the web-based probe designer (https://www.biosearchtech.com/stellaris-designer) (DNA oligonucleotide sequences for *EcR* probe are provided in Supplemental material). The probes were labelled with Quasar-570 dye and were purchased from LGC BioSearch Technologies.

### Cell counting on total cell number

Cell lumens of confocal stacks were detected by training an artificial neural network with the pixel classification software YAPiC (https://yapic.github.io/yapic/) (developed by Christoph Möhl and Manuel Schölling, Image and Data Analysis Facility, Core Research Facilities, DZNE). Training data for background and cell lumen regions were collected on two stacks by using ilastik software [43]. With YAPiC, 80% of the collected training data was used to optimize to a three-dimensional u-shaped convolutional network (using YAPiC option "unet_multi_z"). Remaining 20% of label data was used for performance validation. The network was trained over 5000 epochs (48 training steps per epoch) and the weights with highest validation performance (i.e. lowest loss value) were finally selected. The trained classifier was applied to all confocal stacks. Resulting cell lumen image stacks were imported to Imaris software (Bitplane AG) to detect cell objects with the 'Spot' function. The spot size (puncta diameter) was set at 3 μm as average of direct measurements of the images. Background subtraction was selected. The threshold was manually adjusted (usually within 10% among different samples) with visual inspection to ensure that all cells were identified, minimal background staining or noise was recognized, and that individual cells were not double-counted. False-positive spots were manually eliminated. To ensure that user-introduced noise in threshold adjustment did not add significant bias, each image was quantified a second time, at a later time, without access to the prior quantification. If the difference exceeded 20%, the sample was discarded.

### Quantification and statistical analysis

Fisher’s exact test was performed in Microsoft Excel for Mac 2011. Unpaired two tailed *t*-test was performed using Graph-Pad Prism 7 software.

## Supporting information

S1 Fig TGF-β signalling does not control MB neuron survival. (A-A’) Single confocal section across the cell body cluster of adult MB neurons as representative picture of the counting procedure. Cells are marked in white. Green: GAL4*-OK107*-driven mGFP in A,A’. (B) Quantification of number of adult MB neuron cell bodies from *OK107>luciferase RNAi* and *OK107>babo* RNAi brains. Statistical comparison to the control: N.S. Not Significant (two tailed *t* test). (C,D) Adult MB lobes from *OK107>p35* (C) and *OK107>dSmad2* RNAi, *p35* (D) brains stained with anti-FasII antibody (magenta). Green: GMR26E01*-*LexA-α’β’*-*MB-driven mGFP in C,D. (E) Quantification of number of adult α’β’ MB neuron cell bodies from *OK107>p35* (C), *OK107>dSmad2* RNAi, *p35* (D) and *OK107> dSmad2* RNAi brains. Statistical comparison to the control: ***, p<0.001 (two tailed *t* test); N.S. Not Significant.
(DOCX)

S2 Fig Blocking TGF-β signalling does not affect larval MB neuron gross morphology and identity. (A-C) WL3 MB lobes from control (A) (n = 10), *babo*^*9*^ (B) (n = 14) and *dSmad2*^*1*^ (C) (n = 13) MARCM NB clones induced at NHL and labelled with mGFP (green) using the GAL4-*OK107* driver and stained with anti-FasII antibody (magenta). (D-I’) WL3 MB lobes of control (D,D’, G,G’), *OK107>babo* RNAi (E,E’, H,H’), *OK107>dSmad2* RNAi (F,F’, I,I’) brains visualized with GAL4*-OK107*-driven mGFP (green) and labelled with anti-FasII (D-F’) or Trio (G-I’) antibodies (magenta).
(DOCX)

S3 Fig TGF-β signalling is dispensable for the expression of the fate determinants Chinmo and Abrupt. (A-D’) Cell bodies of control (A,A’, C,C’) and *babo*^*9*^ (B,B’, D,D’) MARCM MB neuroblast clones (white dashed line) induced at NHL and analysed at WL3. MARCM MB clones visualized with mGFP (green) expressed by the GAL4*-OK107* driver and labelled with anti-Chinmo (A-B’) or Abrupt (C-D’) antibodies (magenta).
(DOCX)

S4 Fig The expression of Chinmo is required for TGF-β signalling-dependent MB fate specification. (A-D) Adult MB lobes from control (A), *chinmo*^*1*^ (B), *chinmo*^*1*^; *UAS-babo-CA* (C) and *chinmo*^*1*^; *UAS-dSmad2-CA* (D) neuroblast MARCM clones generated at mid-3rd instar, labelled with mGFP (green) using the GAL4-*OK107* driver and stained with anti-FasII antibody (magenta). (E) Quantification of α’β’ MB fate defects in control, *chinmo*^*1*^, *chinmo*^*1*^; *UAS-babo-CA* and *chinmo*^*1*^; *UAS-dSmad2-CA* neuroblast clones. Statistical comparison to *chinmo*^*1*^: N.S. Not Significant (Fisher’s exact test). (F-I’) Cell bodies of control (F, F’), *chinmo*^*1*^ (G, G’), *chinmo*^*1*^; *UAS-babo-CA* (H, H’) and *chinmo*^*1*^; *UAS-dSmad2-CA* (I, I’) MARCM MB neuroblast clones (white dashed line) induced at NHL and analysed at WL3. MARCM MB clones were visualized with mGFP (green) expressed by the GAL4*-OK107* driver and labelled with anti-EcR-B1 antibody (magenta).
(DOCX)

S5 Fig Postmitotic EcR-B1 expression in WL3 MB neurons. MB progenitors cell bodies from *worniu>*mGFP (green) brains at WL3, co-labelled with anti-EcR-B1 antibody (magenta).
(DOCX)

S6 Fig EcR-B1 expression in MB precursors does not rescue TGF-β signalling-dependent fate specification defects. (A, B) Adult MB lobes from *worniu>EcR-B1* (A) and *worniu>babo* RNAi, *EcR-B1* (B) brains stained with anti-FasII antibody (magenta). Green: GMR26E01*-*LexA-α’β’*-*MB-driven mGFP in A,B. (C) Quantification of number of adult α’β’ MB neuron cell bodies from *worniu>EcR-B1* and *worniu>babo* RNAi, *EcR-B1* brains. Statistical comparison to the control: ***, p<0.001 (two tailed *t* test). (D-E) WL3 MB cell bodies from *worniu>EcR-B1* (D) and *worniu>babo* RNAi, *EcR-B1* (E) brains stained with anti-EcR-B1 antibody (magenta). Anti-Abrupt (blue) staining was used as landmark in locating MB neurons.
(DOCX)
