# Supplementary figures and images for: Modulators of hormonal response regulate temporal fate specification in the Drosophila brain
Source: PLoS Genet. 2019 Dec 6;15(12):e1008491. doi: 10.1371/journal.pgen.1008491 (PMC6919624; doi:10.1371/journal.pgen.1008491)

#### SUPPLEMENTAL FIGURE 1


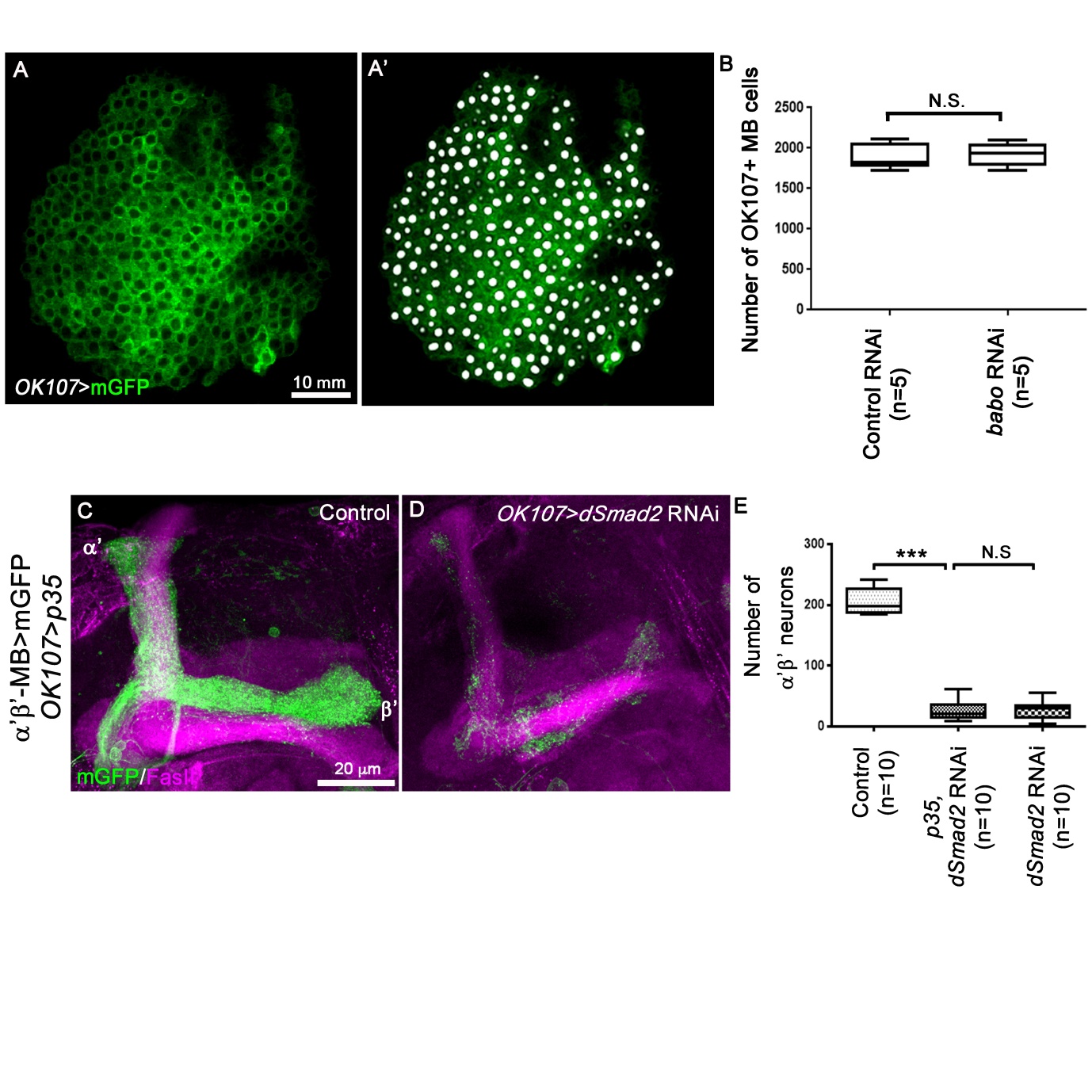

Supplement: S1 Fig — (A-A’) Single confocal section across the cell body cluster of adult MB neurons as representative picture of the counting procedure. Cells are marked in white. Green: GAL4-OK107-driven mGFP in A,A’. (B) Quantification of number of adult MB neuron cell bodies from OK107>luciferase RNAi and OK107>babo RNAi brains. Statistical comparison to the control: N.S. Not Significant (two tailed t test). (C,D) Adult MB lobes from OK107>p35 (C) and OK107>dSmad2 RNAi, p35 (D) brains stained with anti-FasII antibody (magenta). Green: GMR26E01-LexA-α’β’-MB-driven mGFP in C,D. (E) Quantification of number of adult α’β’ MB neuron cell bodies from OK107>p35 (C), OK107>dSmad2 RNAi, p35 (D) and OK107> dSmad2 RNAi brains. Statistical comparison to the control: ***, p<0.001 (two tailed t test); N.S. Not Significant. (DOCX) [file pgen.1008491.s001.docx]

#### SUPPLEMENTAL FIGURE 3


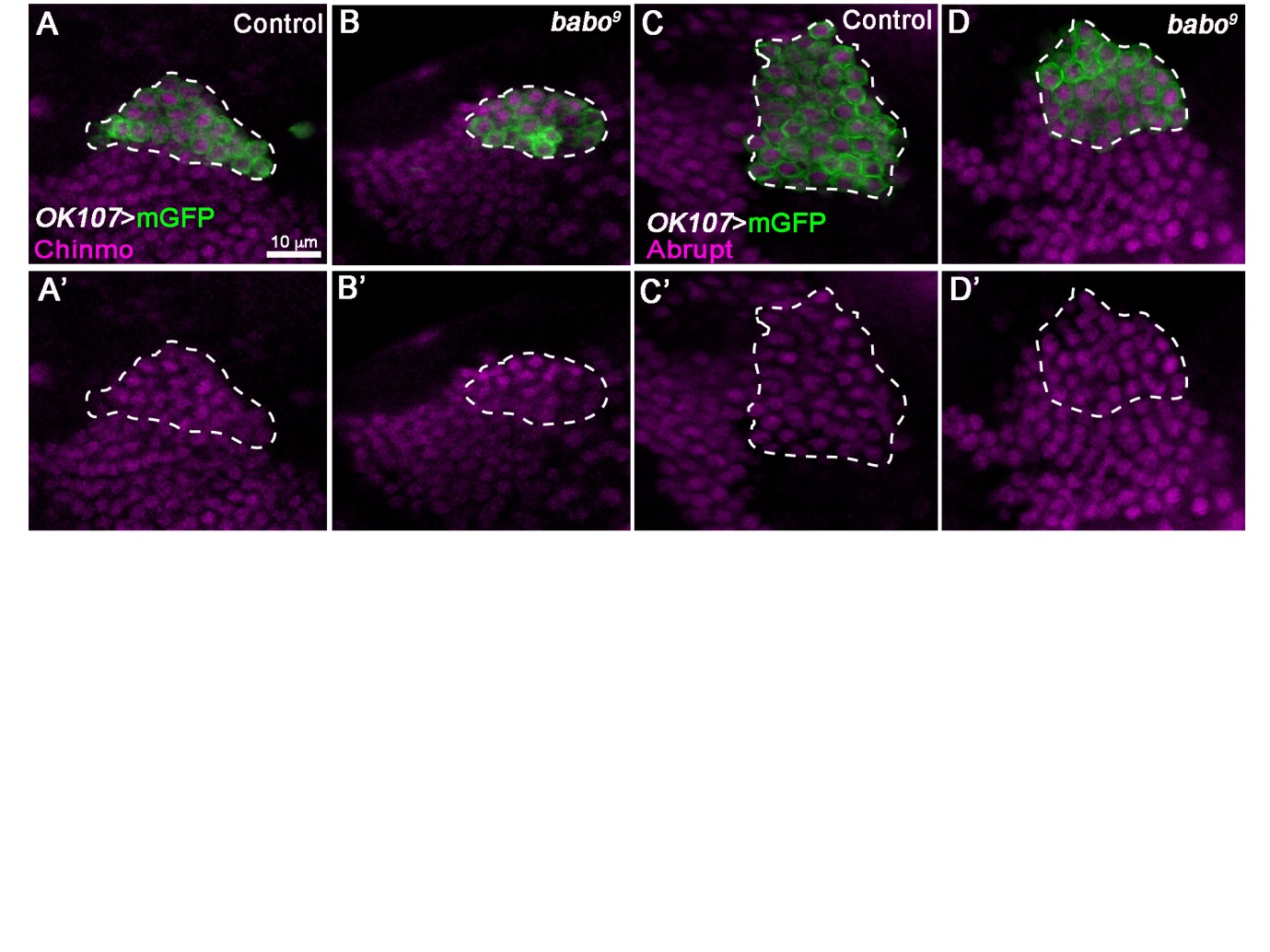

Supplement: S3 Fig — (A-D’) Cell bodies of control (A,A’, C,C’) and babo9 (B,B’, D,D’) MARCM MB neuroblast clones (white dashed line) induced at NHL and analysed at WL3. MARCM MB clones visualized with mGFP (green) expressed by the GAL4-OK107 driver and labelled with anti-Chinmo (A-B’) or Abrupt (C-D’) antibodies (magenta). (DOCX) [file pgen.1008491.s003.docx]

#### SUPPLEMENTAL FIGURE 4


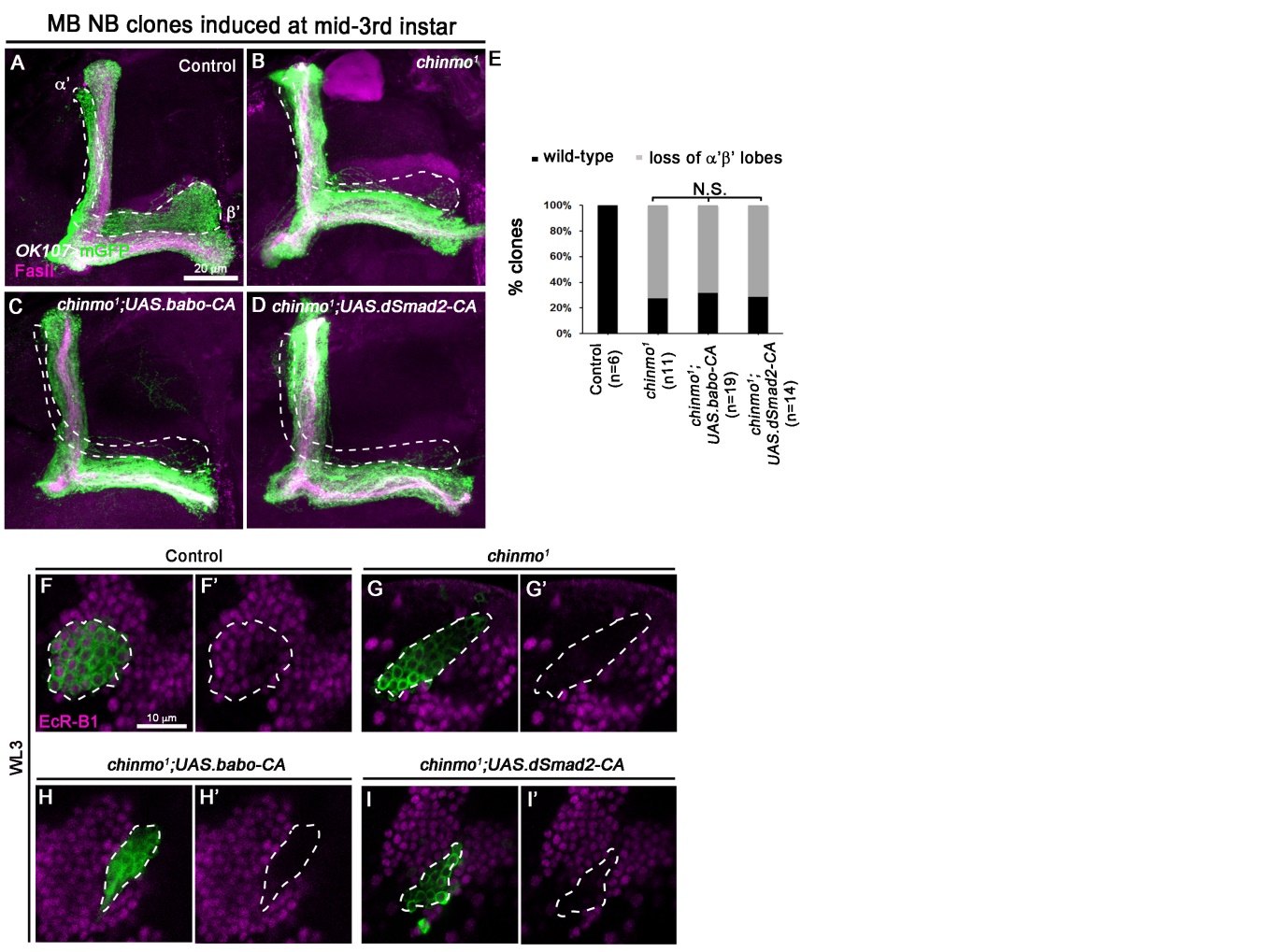

Supplement: S4 Fig — (A-D) Adult MB lobes from control (A), chinmo1 (B), chinmo1; UAS-babo-CA (C) and chinmo1; UAS-dSmad2-CA (D) neuroblast MARCM clones generated at mid-3rd instar, labelled with mGFP (green) using the GAL4-OK107 driver and stained with anti-FasII antibody (magenta). (E) Quantification of α’β’ MB fate defects in control, chinmo1, chinmo1; UAS-babo-CA and chinmo1; UAS-dSmad2-CA neuroblast clones. Statistical comparison to chinmo1: N.S. Not Significant (Fisher’s exact test). (F-I’) Cell bodies of control (F, F’), chinmo1 (G, G’), chinmo1; UAS-babo-CA (H, H’) and chinmo1; UAS-dSmad2-CA (I, I’) MARCM MB neuroblast clones (white dashed line) induced at NHL and analysed at WL3. MARCM MB clones were visualized with mGFP (green) expressed by the GAL4-OK107 driver and labelled with anti-EcR-B1 antibody (magenta). (DOCX) [file pgen.1008491.s004.docx]

#### SUPPLEMENTAL FIGURE 5


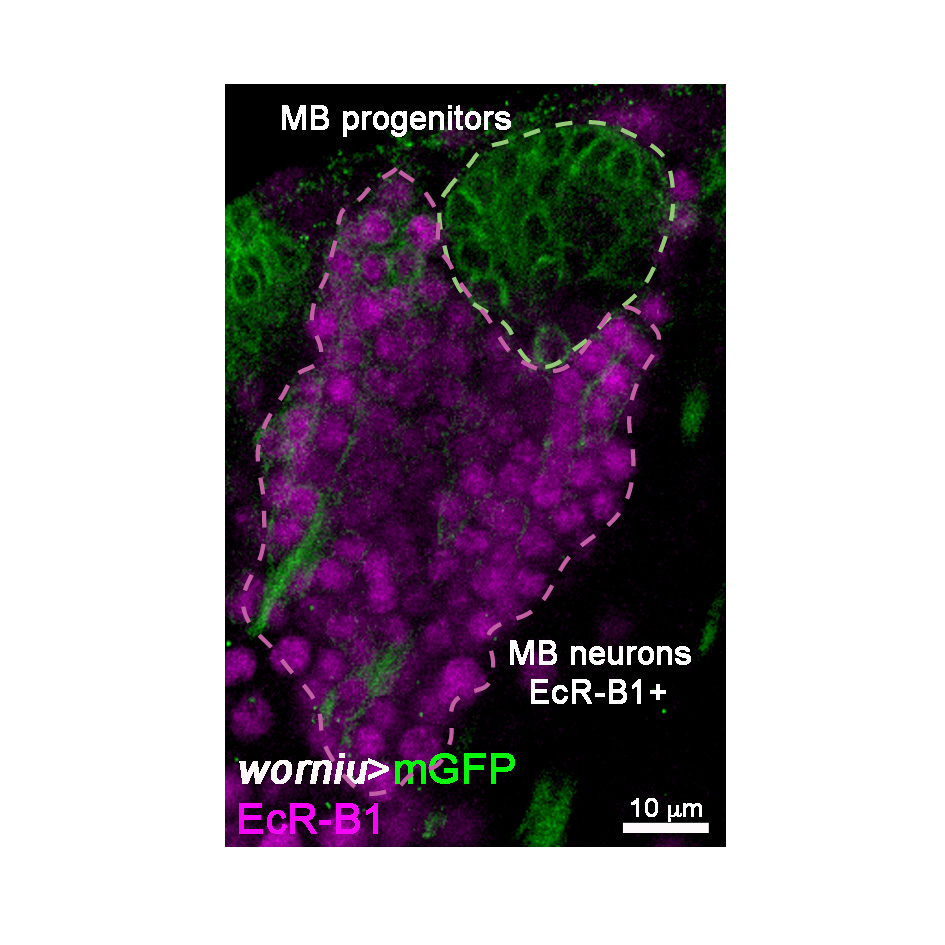

Supplement: S5 Fig — MB progenitors cell bodies from worniu>mGFP (green) brains at WL3, co-labelled with anti-EcR-B1 antibody (magenta). (DOCX) [file pgen.1008491.s005.docx]

#### SUPPLEMENTAL FIGURE 6


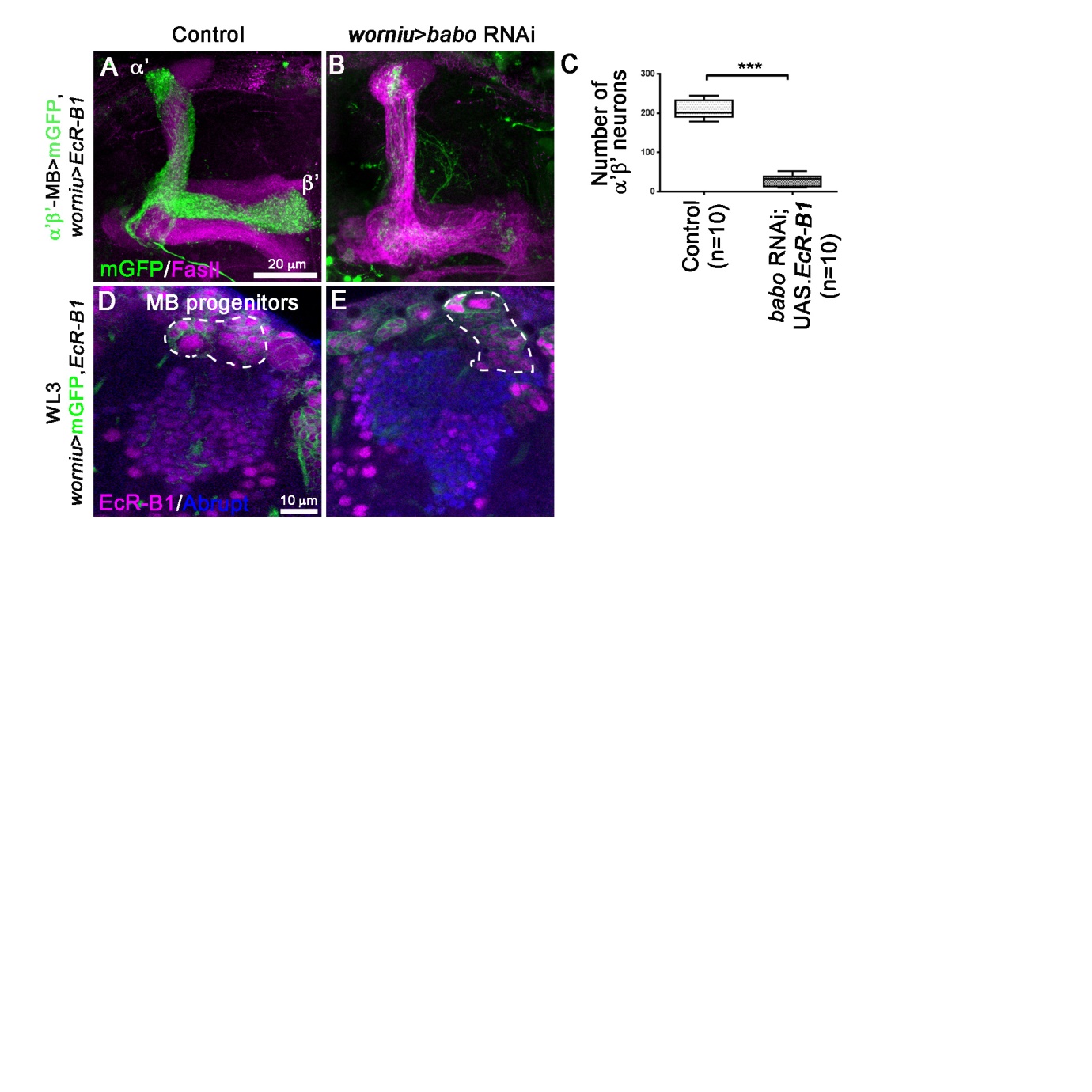

Supplement: S6 Fig — (A, B) Adult MB lobes from worniu>EcR-B1 (A) and worniu>babo RNAi, EcR-B1 (B) brains stained with anti-FasII antibody (magenta). Green: GMR26E01-LexA-α’β’-MB-driven mGFP in A,B. (C) Quantification of number of adult α’β’ MB neuron cell bodies from worniu>EcR-B1 and worniu>babo RNAi, EcR-B1 brains. Statistical comparison to the control: ***, p<0.001 (two tailed t test). (D-E) WL3 MB cell bodies from worniu>EcR-B1 (D) and worniu>babo RNAi, EcR-B1 (E) brains stained with anti-EcR-B1 antibody (magenta). Anti-Abrupt (blue) staining was used as landmark in locating MB neurons. (DOCX) [file pgen.1008491.s006.docx]
