# Supplementary material for: Modulators of hormonal response regulate temporal fate specification in the Drosophila brain
Source: PLoS Genet. 2019 Dec 6;15(12):e1008491. doi: 10.1371/journal.pgen.1008491 (PMC6919624; doi:10.1371/journal.pgen.1008491)
Supplement: S2 Fig — (A-C) WL3 MB lobes from control (A) (n = 10), babo9 (B) (n = 14) and dSmad21 (C) (n = 13) MARCM NB clones induced at NHL and labelled with mGFP (green) using the GAL4-OK107 driver and stained with anti-FasII antibody (magenta). (D-I’) WL3 MB lobes of control (D,D’, G,G’), OK107>babo RNAi (E,E’, H,H’), OK107>dSmad2 RNAi (F,F’, I,I’) brains visualized with GAL4-OK107-driven mGFP (green) and labelled with anti-FasII (D-F’) or Trio (G-I’) antibodies (magenta). (DOCX) [file pgen.1008491.s002.docx]

#### SUPPLEMENTAL FIGURE 2
